# Use of echocardiography in percutaneous closure of patent ductus arteriosus at the Instituto Nacional de Salud del Niño, San Borja, Lima - Peru

**DOI:** 10.47487/apcyccv.v5i2.350

**Published:** 2024-06-24

**Authors:** Alex I. Catalán, Karen Condori, Mónica Medina, Stella Lucena, David Montoya, Ricardo Gálvez-Arévalo

**Affiliations:** 1 Área de Cateterismo Cardíaco Pediátrico - Instituto Nacional de Salud del Niño de San Borja. Lima, Peru. Área de Cateterismo Cardíaco Pediátrico Instituto Nacional de Salud del Niño de San Borja Lima Peru; 2 Instituto Nacional de Salud del Niño de San Borja. Lima, Peru. Instituto Nacional de Salud del Niño de San Borja Lima Peru; 3 Área de Cuidado intensivos, Hospital Regional Virgen de Fátima. Chachapoyas, Peru. Área de Cuidado intensivos Hospital Regional Virgen de Fátima Chachapoyas Peru; 4 Sub Unidad de Investigación e Innovación Tecnológica, Instituto Nacional de Salud del Niño de San Borja. Lima, Peru. Sub Unidad de Investigación e Innovación Tecnológica Instituto Nacional de Salud del Niño de San Borja Lima Peru

**Keywords:** Echocardiography, Ductus Arteriosus, Patent, Fluoroscopy, Ecocardiografía, Conducto Arterioso Persistente, Fluoroscopía

## Abstract

**Objetive.:**

Percutaneous occlusion of patent ductus arteriosus (PDA) has classically been performed entirely by fluoroscopy, however in recent years, transthoracic echocardiography (TE) has been used as an aid to fluoroscopy or entirely by echocardiography, which avoids access of femoral artery, use of contrast and decrease in time and dose of radiation exposure. The objective of this study was to evaluate the success rate with the use of TE in percutaneous PDA closure.

**Material and method.:**

Descriptive, comparative, retrospective study between patients in whom PDA closure was performed with fluoroscopy plus angiography (group 1) and fluoroscopy plus ET (group 2), between January 2018 and December 2022. The data were obtained from the clinical history electronic and procedure report.

**Results.:**

One hundred eight patients were analyzed, fluoroscopy group (n: 57) and TE (n: 51). The success rate in PDA occlusion using TE was 100% and 98% for the fluoroscopy group, with no statistically significant difference The average age of group 2 was 2.9 years, while the average age of group 1 was 5 years (p=0.001), the average fluoroscopy time in group 1 was 16.9 min and 4.71 min in group 2 (p < 0.001); the fluoroscopy dose in group 1 was 68.98 mGy and 5.17 mGy in group 2 (p<0.001). Krichenko, but without significant difference in both groups.

**Conclusions.:**

The success rate of percutaneous PDA closure using echocardiography and fluoroscopy is appropiate, with a success rate similar to the classic technique. In addition, it makes it possible to reduce the dose and time of fluoroscopy, avoid the use of contrast, and access the femoral artery.

## RESUMEN

**Objetivo.:**

La oclusión percutánea del ductus arterioso persistente (DAP) clásicamente se ha realizado por fluoroscopía y angiografía; sin embargo, en los últimos años se está utilizando la ecocardiografía transtorácica (ETT) como ayuda a la fluoroscopía o íntegramente por ETT, lo que evita el acceso de arteria femoral, uso de contraste y disminución de tiempo y dosis exposición a la radiación. El objetivo del estudio fue evaluar la tasa de éxito con la utilización de la ETT en el cierre percutáneo del DAP.

**Materiales y métodos.:**

Estudio descriptivo de tipo comparativo, retrospectivo, entre pacientes en quienes se realizó el cierre del DAP con fluoroscopía más angiografía (grupo 1) y fluoroscopía más ETT (grupo 2), entre enero 2018 y diciembre 2022. Los datos fueron obtenidos de la historia clínica electrónica y del informe del procedimiento.

**Resultados.:**

Se analizaron 108 pacientes, de los cuales 57 pertenecen al grupo de fluoroscopía más angiografía y 51 al grupo de fluoroscopía más ETT. La tasa de éxito en la oclusión del DAP utilizando fluoroscopía más ETT fue del 100% y 98% para el grupo de fluoroscopía más angiografía, sin diferencia estadísticamente significativa. La edad promedio del grupo 2 fue de 2,9 años, mientras que la edad promedio del grupo 1 fue 5 años (p< 0,001), el tiempo promedio de fluoroscopía en el grupo 1 fue de 16,9 min y 4,7 min en el grupo 2 (p < 0,001); la dosis de fluoroscopía en el grupo 1 fue de 68,98 mGy y 5,17 mGy en el grupo 2 (p< 0,001). Se encontró que el tipo de DAP predominante fue el tipo A de Krichenko, pero sin diferencia significativa en ambos grupos.

**Conclusión.:**

La tasa de éxito del cierre percutáneo del DAP con el uso de la ecocardiografía y fluoroscopia es adecuada, con un nivel de éxito similar a la técnica clásica, esto permite, además, disminuir la dosis y tiempo de fluoroscopia, evitar el uso de contraste y el acceso de la arteria femoral

## Introduction

In utero, the ductus arteriosus is permeable and carries 85% of the cardiac output ejected by the right ventricle, where gas exchange occurs in the placenta. After birth, gas exchange takes place in the lungs. The increase in oxygen saturation along with the decrease in circulating prostaglandins leads to the closure of the ductus within the first 48 h. Pulmonary pressure begins to drop, and spontaneous closure of the ductus arteriosus occurs over the following weeks. It is considered that by 8 weeks, the duct is closed in 80% of children ^1^^,^^2^.

In Peru, patent ductus arteriosus (PDA) is the second most common acyanotic congenital heart disease among children under one year of age, following ventricular septal defect ^3^. It is known that certain factors, such as a history of prematurity ^1^, genetic syndromes ^4^, or the altitude above sea level from which the patient originates ^5^, predispose to the PDA. Depending on the size and age of the patient, there may be symptoms of congestive heart failure ^6^, risk of pulmonary hypertension, risk of endarteritis, and even reports of aneurysm of the ductus arteriosus ^7^.

Once PDA is diagnosed, it is determined whether surgical or interventional measures are required ^8^. The classic technique for percutaneous closure of the PDA involves fluoroscopy and angiography in the hemodynamics room ^9^. However, considering that children are more sensitive to radiation compared to adult patients and have a longer life expectancy, the risk of developing radiation-attributable diseases increases over their lifetime. Therefore, finding new techniques that help reduce radiation doses will benefit not only the patients but also the operators ^10^^,^^11^.

In recent years, percutaneous closure of PDA using transthoracic echocardiography (TTE) has been reported ^12^^,^^13^. This approach avoids femoral artery puncture, reduces or eliminates the need for fluoroscopy, and avoids the use of contrast agents, taking into account that some patients may have underlying renal comorbidities ^14^. There is a knowledge gap regarding the reporting of the use of echocardiography in Latin American hospitals. Therefore, the main objective of this study was to evaluate the success rate of the percutaneous closure of the ductus arteriosus using echocardiography in the pediatric population treated at the Instituto Nacional de Salud del Niño San Borja in Lima - Peru.

## Materials and methods

### Study design and population

This is a retrospective, observational, comparative study. Electronic medical records and procedure reports of patients aged 0 to 18 years who underwent percutaneous closure of the PDA from January 2018 to December 2022 were reviewed. The study included 108 patients with a weight over 3 kg and a PDA that showed signs of hemodynamic impact in clinical and echocardiographic evaluation. Patients with poor transthoracic windows, those with other associated heart conditions requiring surgery, and those with incomplete data in the medical record or procedure report were excluded. The patients were divided into two groups: those whose PDA closure was performed using fluoroscopy and angiography (Group 1) and those whose closure was done using fluoroscopy and TTE (Group 2).

### Procedure

The indication for percutaneous closure of the PDA was made after clinical discussion in a cardio-surgical board meeting. During the first two years of the registry, the procedure was performed exclusively using the classic technique with fluoroscopy and angiography. However, in the last two years of the registry, fluoroscopy combined with TTE was also incorporated, taking into account the inclusion and exclusion criteria. Prior to the procedure, all patients underwent a TTE with supraesternal and left parasternal views to assess the shape and size of the PDA, as well as to confirm that there were no other cardiac lesions.

The procedure was carried out in the hemodynamics room using a Siemens Artis Zee biplane cineangiograph and an Aloka Pro Sound Alpha 7 echocardiograph, with the patient intubated and under general anesthesia, and with aseptic and antiseptic preparation of the inguinal areas. Only two pediatric cardiology specialists were involved in all procedures for both groups. In Group 1, both arterial and venous access were obtained using the Seldinger technique, whereas in Group 2, only venous access was performed. Short 4 or 5 Fr introducers were used, and after vascular access was established, 100 IU/kg of unfractionated heparin (UFH) was administered.

**Figure 1.**
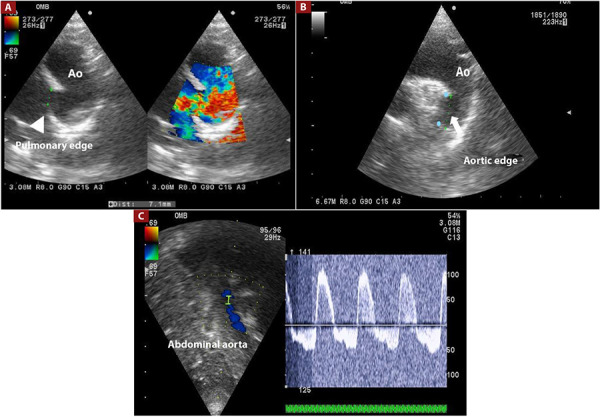
(A) Left parasternal view for the measurement of the pulmonary end of the PDA, (B) Left parasternal view for the measurement of the aortic end of the PDA, (C) Doppler spectrum at the abdominal aorta indicating the impact of the PDA. PDA: patent ductus arteriosus

In patients from Group 1, a 4 or 5 Fr Pigtail catheter was used for aortography in the left lateral 90°/0° and right anterior oblique 30°/0° projections, which allowed for the confirmation of the anatomy and dimensions of the PDA. In Group 2, the assessment of the PDA’s anatomy was performed using TTE in the supraesternal and left parasternal windows (Figures 1A and B). In Group 2, Doppler spectra through the PDA and in the abdominal aorta were also recorded (Figure 1C) ^12^^,^^13^.

The devices used were ductus occluders from Abbot, Oclutech, Memopart and Liftech Medical. Other devices used were multifunctional devices (Liftech Medical), ventricular septal defect occluders (Liftech Medical), vascular occluders II (Abbot), Nit-Occluder (PF Medical), and PCA coils (Cook Medical). The size of the PDA occluder device used in Group 1 was ≥ 2 mm and in Group 2 it was ≥ 4 mm, based on the measurement taken at the narrowest part of the patent ductus arteriosus.

In both groups, access to the PDA was achieved through the femoral vein using either a 4/5 Fr multipurpose catheter or a 4/5 Fr right coronary catheter, depending on the case. The catheter was guided through the right atrium, right ventricle, pulmonary trunk, PDA, and descending aorta under fluoroscopic guidance. In all cases, pulmonary arterial pressure and aortic pressure were measured.

Subsequently, in all patients, either the multipurpose catheter or the right coronary catheter was positioned in the descending aorta using a 0.035” x 260 cm hydrophilic guide, and was exchanged for the extra-support guide 0.035” x 260 cm, which was positioned in the thoracoabdominal aorta. Following this, the short 4/5 Fr introducer was swapped for a longer introducer compatible with the occlusion device to be used. The dilator of the long introducer and the extra-support guide were then removed, and the PDA occluder device was introduced.

**Figure 2:**
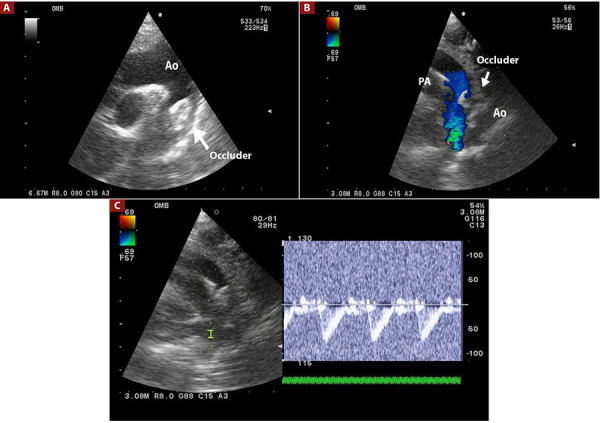
(A) Device approaching the aortic end of the PDA. (B) Device fully deployed in the PDA, not yet released, with no evidence of residual shunt, no aortic obstruction, and no obstruction of the left pulmonary branch. (C) Doppler spectrum in the left pulmonary branch with no evidence of obstructive gradient.

In Group 1, angiographic controls were performed in lateral 90°/0° and left anterior oblique 30°/0° projections for the positioning of the occluder, assessing for residual shunt and potential aortic obstruction. In Group 2, the occluder was positioned using TTE in the supraesternal and left parasternal windows, evaluating for residual shunt, aortic flow, and left pulmonary branch flow (Figures 2A, B and C). The procedure concluded by releasing the occluder, removing the delivery system and the long introducer, and performing compressive hemostasis at the puncture site.

### Study variables

The study included the success rate defined as the number of successful procedures relative to the total number of ductus closure procedures in each group. Both groups were compared based on various parameters including age, sex, weight, altitude of origin, genetic syndrome, comorbidity associated at the time of procedure, functional class, morphology of the PDA, pulmonary end measurement, pulmonary pressure, fluoroscopy time and fluoroscopy dose (measured in milligray - mGy), which is defined as the energy of X-rays absorbed by the patient and operator, use of contrast, presence of device embolisation, puncture site thrombosis, residual shunts and aortic obstruction.

### Statistical analysis

To compare categorical variables, the chi-square test was used, and for quantitative variables, after verifying the normal distribution of the variables, the Student’s t-test was conducted.

A two-tailed alpha error of 5% was considered statistically significant (p<0.05). Statistical analyses were performed using IBM SPSS Statistics software (version 22, SPSS, IBM Corporation, Armonk, New York).

## Results

A total of 108 patients were analyzed, with 57 in the fluoroscopy plus angiography group (Group 1) and 51 in the fluoroscopy plus TTE group (Group 2). The characteristics of both groups were similar and are described in Table 1.

**Table 1:** Comparison between percutaneous closure of patent ductus arteriosus by fluoroscopy and transthoracic echocardiography.

	Group 1 (n=57)	Group 2 (n =51)	p-value
Age (years) X (SD)	5.09 (4.3)	2.96 (2.4)	0.001
Weight (kg) X (SD)	18.95 (12.4)	12.18 (6.7)	< 0.001
Female Sex	42 (56 %)	33 (44%)	0.427 *
Origin Altitude (masl)			0.115*
< 1000	38 (57.58%)	28(42.4%)	
1000-2500	3(25%)	9 (75%)	
> 2500	16 (53.3%)	14 (46.7%)	
Genetic Syndrome (Down)	5 (8.8%)	12 (23.5 %)	0.066 *
Comorbidity	1 (20.0 %)	4 (80.0 %)	0.296 *
Pulmonary Artery End (mm) X (SD)	3.47 (1.6)	4.04 (1.7)	0.077
Pulmonary Pressure X (SD)	21.93(6.6)	25.02 (9.9)	0.063
Ductus Morphology (Krichenko)^15^			0.058 *
A	51 (50.0 %)	51 (50.0 %)	
C	2 (100.0 %)	0 (0.0 %)	
E	4 (100.0 %)	0 (0.0 %)	
Functional Class (Ross)^6^			< 0.003 *
I	30 (74.0 %)	11 (26.0 %)	
II	22(38.6 %)	35 (61.5 %)	
III	5 (50.0 %)	5 (50.0 %)	
Fluoroscopy Time (min) X (SD)	16.91 (5.9)	4.71 (3.5)	< 0.001
Fluoroscopy Dose (mGy) X (SD)	68.98 (57.2)	5.18 (6.7)	< 0.001

X: mean, SD: standard deviation, masl: meters above sea level.

Group 1: fluoroscopy + angiography, group 2: fluoroscopy + transthoracic echocardiography.

Only for the first two patients in Group 2 was arterial access and angiography also performed to compare the measurement obtained with echocardiography. The size of the occluder device used in Group 1 was ≥ 2 mm and in Group 2, a device ≥ 4 mm at the narrowest part.

The mean age of the patients in Group 2 was significantly younger (5.09 ± 4.3 *vs.* 2.96 ± 2.4, p=0.001); the mean weight was also significantly lower in Group 2 (18.95 ± 12.4 *vs.* 12.18 ± 6.7, p<0.001). There was a female predominance in both groups; however, no significant difference was found (p=0.115).

Most patients in both groups came from altitudes below 1000 meters above sea level (masl); however, one third of the patients came from altitudes greater than 2500 masl, with no significant difference between the groups (p=0.115).

Both groups had patients with Down’s syndrome, 8.8% in group 1 and 23.5% in group 2; however, no significant differences were found between groups (p=0.066).

Among the comorbidities in Group 2, one patient had a history of left pulmonary agenesis, and another had right diaphragmatic paralysis, with no complications during the procedure. In Group 1, two patients were admitted for pneumonia that required prolonged intubation; however, the procedure was carried out without any incidents.

The mean pulmonary pressure with fluoroscopy plus TTE was 25 mmHg *vs.* 21 mmHg in the fluoroscopy and angiography group, with no significant difference (p=0.077). In both groups the most frequent form of ductus arteriosus was Krichenko type A ^15^, with no significant differences (p=0.058); (Table 1).

The fluoroscopy time was significantly shorter for patients undergoing TTE (4.71 ± 3.5 mGy *vs.* 16.91 ± 5.8 mGy, p<0.001). Fluoroscopy dose was also significantly lower in patients with TTE (5.18 ± 6.7 mGy *vs.* 68.98 ± 57.2 mGy, p<0.001).

The success rate for percutaneous closure of the PDA was 100% in the TTE plus fluoroscopy group and 98% in the fluoroscopy plus angiography group. There was one instance of device migration in the fluoroscopy and angiography group, while no migrations were reported in the fluoroscopy and TTE group during the analyzed period, with no significant differences between the two groups. The device migration occurred in one patient due to underestimation of the size of the narrowest part of the ductus arteriosus. In this case, percutaneous extraction of the device was not possible, necessitating surgical removal of the device and ligation of the ductus arteriosus.

Trivial immediate residual shunting was observed in 96% of the patients. However, no evidence of residual shunting was found during the echocardiographic control conducted one week after the procedure.

In both groups, there were no complications in the femoral arterial and venous access areas, and the patients were discharged the day after the procedure, except for two cases that had respiratory comorbidities. Echocardiographic control conducted a week after the procedure showed no evidence of residual shunt in either group. The patients were followed up for at least one year, during which they showed a decrease in cardiac silhouette, no residual shunt, progressive improvement in functional class, and no need for medication.

## Discussion

In our study, we found that the use of TTE reduced the procedure time, eliminated the need for femoral arterial access, and avoided the use of contrast agents. It also minimized radiation exposure, benefiting both patients and operators without compromising the effectiveness of defect closure, as supported by scientific literature ^11^^-^^13^. TTE has also been successfully used for interventricular and atrial septal defects with the same objective of reducing radiation doses and decreasing the need for angiographies ^16^^,^^17^.

In our study, patients who underwent TTE had a mean weight of 12 kg and a mean age of 3 years. This contrasts with the study conducted by Cheng Wang *et al.*^12^, where the mean weight was over 25 kg and the mean age was over 10 years. This suggests that TTE can be effectively used in younger children, considering the good transthoracic window that most of them possess.

Similarly to what was reported by Dimas *et al.*^18^, we had three patients under 6 kg who underwent procedures with fluoroscopy and four with transthoracic echocardiography without any complications. Our study corroborates that the smaller the patients, the generally better the transthoracic window, allowing for excellent visualization of the cardiac structures during the procedure.

Although we did not find significant differences, the literature suggests that patients from high altitudes (>2500 masl) tend to have larger ductus arteriosus ^(5, 19, 20)^, which may even require the use of larger duct occluder devices, up to 14/16 mm in some cases. The lack of detection of differences in our study could be due to the fact that this comparison was not the primary objective, which might have affected the sample size needed to identify differences, if any existed.

Type A ductus, according to Krichenko’s classification ^15^, is the most frequent in both groups in our study, so, considering the advantages already mentioned with the use of transthoracic echocardiography, it would be of great benefit for both the patient and the operators

The use of transthoracic echocardiography offers the advantage of being able to display the characteristics and measurements of the patent ductus arteriosus, the left pulmonary artery, the aorta, as well as color and spectral Doppler imaging. It also allows for adjustments to be made to the device in the ductus arteriosus area and can detect any residual shunt ^21^.

Femoral venous access leads to a quicker procedure as it reduces compression time, anesthesia duration, and speeds up post-anesthesia recovery, all of which benefit the patients.

In conclusion, percutaneous closure of ductus arteriosus using TTE and fluoroscopy is safe and achieves a success rate similar to the classic technique that uses fluoroscopy and angiography. It allows for the use of venous access only, and reduces both the dose and duration of radiation exposure, benefiting both patients and operators. Additionally, it shortens the procedure time and eliminates the need for contrast.

Therefore, the use of transthoracic echocardiography and fluoroscopy is a valid strategy compared to fluoroscopy and angiography, without increasing the complications that can occur during the procedure.
